# Who Adopts Improved Fuels and Cookstoves? A Systematic Review

**DOI:** 10.1289/ehp.1104194

**Published:** 2012-02-01

**Authors:** Jessica J. Lewis, Subhrendu K. Pattanayak

**Affiliations:** 1 Nicholas School of the Environment,; 2 Sanford School of Public Policy, and; 3 Global Health Institute, Duke University, Durham, North Carolina, USA

**Keywords:** adoption regressions, fuel choice, fuel switching, improved cookstove, indoor air pollution, systematic review

## Abstract

Background: The global focus on improved cookstoves (ICSs) and clean fuels has increased because of their potential for delivering triple dividends: household health, local environmental quality, and regional climate benefits. However, ICS and clean fuel dissemination programs have met with low rates of adoption.

Objectives: We reviewed empirical studies on ICSs and fuel choice to describe the literature, examine determinants of fuel and stove choice, and identify knowledge gaps.

Methods: We conducted a systematic review of the literature on the adoption of ICSs or cleaner fuels by households in developing countries. Results are synthesized through a simple vote-counting meta-analysis.

Results: We identified 32 research studies that reported 146 separate regression analyses of ICS adoption (11 analyses) or fuel choice (135 analyses) from Asia (60%), Africa (27%), and Latin America (19%). Most studies apply multivariate regression methods to consider 7–13 determinants of choice. Income, education, and urban location were positively associated with adoption in most but not all studies. However, the influence of fuel availability and prices, household size and composition, and sex is unclear. Potentially important drivers such as credit, supply-chain strengthening, and social marketing have been ignored.

Conclusions: Adoption studies of ICSs or clean energy are scarce, scattered, and of differential quality, even though global distribution programs are quickly expanding. Future research should examine an expanded set of contextual variables to improve implementation of stove programs that can realize the “win-win-win” of health, local environmental quality, and climate associated with these technologies.

Nearly half of the global population relies on solid fuel, such as biomass, coal, or dung, for their cooking needs (Legros et al. 2009; Rehfuess et al. 2006). Indoor air pollution (IAP) emitted by burning solid fuel indoors in poorly ventilated conditions is responsible for 2 million premature deaths per year, or 3.3% of the global burden of disease, particularly women and children [World Health Organization (WHO) 2009]. The adverse health outcomes are chiefly caused by inhalation of fine soot particles ≤ 2.5 μm in aerodynamic diameter (Smith et al. 2009). In addition to adverse health effects, negative social impacts often result from using traditional biomass stoves. For example, inefficient stoves require more time to cook and gather fuel, a burden usually borne by women and children, which diverts their time from education and income-producing activities.

Local environmental impacts arise from damages to ambient air and local forest ecosystems. Because only a fraction of the IAP is deposited indoors, biomass burning contributes to ambient air pollution (Shindell et al. 2011). Additionally, the unsustainable harvest of fuelwood degrades local forests (Hofstad et al. 2009; Köhlin et al. 2011), sometimes even damaging wildlife habitat and watershed functions and contributing to deforestation (Geist and Lambin 2001).

Cooking with unsustainably harvested biomass can affect climate because inefficient fuel combustion releases products of incomplete combustion with a higher global warming potential than carbon dioxide, such as methane and carbon monoxide (Sagar and Kartha 2007). Biomass and fossil fuel cookstoves also emit 22% and 7% of global black carbon (BC) emissions, respectively, which is the second strongest contributor to current global warming (Ramanathan and Carmichael 2008). Unlike globally distributed greenhouse gases, such as carbon dioxide, the shorter 8- to 10-day atmospheric lifetime of BC results in localized impacts (Smith et al. 2009).

Improved cookstoves (ICSs) were developed initially to address these adverse health and livelihood impacts. Because ICSs improve cooking efficiency compared with a traditional stove, ICSs can reduce the amount of fuel required, fuel gathering time, and cooking time—all of which have the potential to improve health and increase household income. In addition, these efficiencies can benefit the local environment and global climate because of reductions in fuelwood harvesting and particulate emissions. Despite clear scientific evidence on efficacy of these innovations, initial efforts to promote these technologies have run into challenges surrounding diffusion, dissemination, and implementation.

Initially, failed attempts to foist untested technologies on reluctant households and consumers initially turned the focus of research to identifying the drivers of demand. The demand-side of thinking has been bolstered by a small yet growing body of field evidence suggesting that potential consumers often do not invest in or maintain use of environmental health technologies (e.g., piped water, water filters, private latrines, insecticide treated bed nets, improved stoves), because they do not know about or value the benefits of the technology. In addition, consumers are unwilling to finance or unable to pay the prevailing prices for the technologies (Pattanayak and Pfaff 2009). More generally, implementation and diffusion challenges may be due to ICSs that are unsuitable for local customs, ineffective financing, poor distribution channels, or insufficient social marketing (Mitchell 2010).

Several coinciding “game changers” may now make the large-scale deployment of ICSs more feasible: the development of a new generation of ICSs, significant experience in implementing small-scale credit operations, and new financing instruments and sources, especially those linked to climate change mitigation (World Bank 2011). The influence of the game changers is further strengthened by general trends in low-income countries such as the rising cost of fuelwood (because of increasing scarcity and forest sector reforms). Collectively, these forces have led to increased attention on ICSs and related technologies, culminating in the recent formation of the Global Alliance for Clean Cookstoves (GACC; 2011), which aims to have 100 million homes adopt clean cookstoves by 2020. Additionally, countries such as India have launched a new National Biomass Cookstoves Initiative in 2009 to provide 160 million ICSs to households currently using solid biomass fuel (Venkataraman et al. 2010).

To realize all these goals, the international public health community needs *a*) much firmer empirical bases for the many outstanding questions regarding the drivers of adoption and diffusion of ICSs and clean fuels, *b*) improved scientific learning about implementing promotion programs (Madon et al. 2007), and *c*) practice-based evidence of adoption (Green et al. 2009; Martin et al. 2011). The science of adoption cannot afford to focus only on internal validity of a few adoption factors while ignoring contextual drivers (Glasgow et al. 2003). Thus, it is imperative to match ICS types and cooking preferences and to consider the effectiveness of credit, information campaigns, local institutions, and the supply chain. Systematic reviews can serve as a starting point to learn about the broader trends cross-cutting studies—not an idiosyncratic finding unique to a setting or program or study.

Our review shows that the empirical (quantitative) literature base of adoption studies remains narrow, thin, and scattered. The quality of the research varies highly in terms of study design, measurement approaches, statistical analysis, and sample sizes. Furthermore, no studies have taken a systematic approach. In conducting a systematic review, we add to the qualitative analyses of ICS adoption that have discussed the influence of factors such as affordability (Slaski and Thurber 2009), funding source (Bailis et al. 2009), user engagement (Pohekar et al. 2005), technology design that responds to consumer preference (Sinton et al. 2004), and local scarcity of fuelwood and stove manufacture by local artisans (Barnes et al. 1993). Unfortunately, current empirical adoption studies have rarely considered these drivers, focusing instead on income, education, prices, and on household size and composition.

In our systematic review, we organized the literature to identify adoption drivers that are consistent and externally valid and identify knowledge gaps. We join the evidence base with theory to test basic hypotheses about whether demand factors (e.g., education), supply factors (e.g., dynamic organizations), or both offer the potential to attain sustainable and scalable outcomes. By fulfilling these objectives, we can facilitate the design of policies and planning of programs and projects that are touted to deliver the global, local, and household benefits of clean household energy technologies in developing countries.

## Materials and Methods

In this article, we focus on the following question: What factors are associated with household adoption of clean energy in poor countries? We consider empirical studies of ICS adoption or clean fuel choice (a movement away from solid fuel) and review empirical studies to both frame the overall questions and interpret the findings. Our decision to focus on the adoption of ICSs and clean fuel draws on a decade-old persistent literature in epidemiology and public health, and more recently in environmental and climate science, that highlights the role of household choices relating to choice of stoves and fuels (Bruce et al. 2000; Ezzati et al. 2004; GACC 2011; Martin et al. 2011; Mitchell 2010; Smith et al. 2004, 2011; WHO 2009; World Bank 2011). Given where the IAP problem is centered, we considered households in poor countries that could use ICSs, biogas, kerosene, liquefied petroleum gas (LPG), electricity, and renewable energy sources.

Adoption of ICSs should not imply that households abandon their traditional cookstove (Ruiz-Mercado et al. 2011). Rather, “adoption” within this article represents some use of an ICS. Similarly, by describing clean fuel choice as a movement “up the energy ladder,” we do not mean to imply that households use cleaner fuels exclusively (Farsi et al. 2007; Gundimeda and Köhlin 2008; Pine et al. 2011), but rather that they start to use at least some “cleaner” fuel (Masera et al. 2000). We therefore find “fuel choice” to be a more accurate term than “fuel switching.” As with ICS adoption, partially switching away from animal dung or crop residue to wood, charcoal, kerosene, coal, LPG, and electricity can also have dramatic health, environmental quality, and climate benefits.

We employ the simplest form of a systematic review: vote counting, in which the reviewer categorizes associations (e.g., between adoption and education) as significantly positive, significantly negative, or not significant for each variable. For each variable, each analysis therefore casts a “vote” in support of one of the three types of relationship—positive, negative, or not significant—with the level of significance recorded. Thus, a count of the votes across the studies suggests a general relationship for that specific variable (e.g., education). The analysis is repeated for all indicators of interest. As such, vote counting provides a useful starting point for a systematic assessment of studies within a given research area, and this methodology has been popular in medicine (Cook et al. 1992; Hölzel et al. 2011), natural resource management (Beach et al. 2005; Pattanayak et al. 2003), and public health (Flodgren et al. 2011; Gerrard et al. 1996).

*Search strategy.* To be eligible for inclusion, we selected studies that *a*) considered the use of ICSs and/or clean fuels as an outcome, *b*) used multivariate regression analysis, *c*) included at least two determinants from socioeconomic, physiographic, market, or institutional domains, *d*) treated the household as the unit of analysis, and *e*) sampled populations from a developing country.

Initially, two student groups conducted literature reviews under the supervision of one author (S.K.P.). Subsequently, both authors revised the search strategy and the inclusion and exclusion criteria, one author (J.J.L.) extracted data and performed the synthesis, and the other author (S.K.P.) reviewed the extraction results, synthesis, and interpretations. The search of three major databases, ScienceDirect (2011), Google Scholar (2011), and ISI Web of Science (Thomson Reuters 2011), was conducted between 7 February and 20 June 2011. Our search algorithm considered permutations and combinations of keywords, grouped by category. The algorithm used for the ISI Web of Science search is provided in the Supplemental Material (http://dx.doi.org/10.1289/ehp.1104194). Algorithms included a term from each of the following categories: *a*) fuel (“cookstove,” “biomass,” “fuelwood,” “fuel wood,” “firewood,” “biogas,” “electricity,” “solar power,” “photovoltaic,” “renewable,” “charcoal,” “energy,” “energy ladder”); *b*) choice (“choice,” “choos*,” “switch*,” “adoption,” “dissemination,” “uptake”); *c*) scale (“household,” “residential,” “domestic”); and *d*) analysis method (“regression,” “statistics”).

To address publication bias, we also sought out and reviewed unpublished and gray literature, particularly local research, from Asia, Central America, and Africa provided by household-energy experts. We did not exclude any article based on country or language of publication.

**Figure 1:**
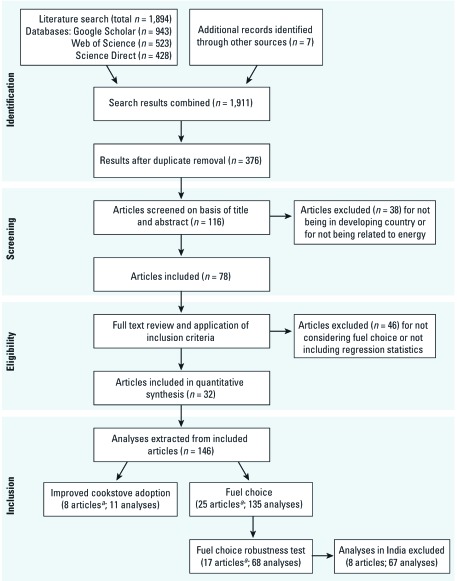
PRISMA (Preferred Reporting Items for Systematic Reviews and Meta-analysis) flow diagram for searching and extracting data (adapted from Moher et al. 2009). ***^a^***One article contained both ICS and fuel choice analyses.

*Data extraction.* This search yielded 1,911 papers (see Figure 1), of which 32 met our inclusion criteria (Table 1). From these 32 papers, a total set of 146 separate analyses were extracted for the systematic review. The high number of analyses from a small set of studies is a result of multiple regression analyses within single studies (e.g., comparing choice of kerosene over biomass and LPG over biomass and conducting these analyses for rural and urban locations separately).

**Table 1:** Studies included in systematic review.

Reference	Country	Fuel choice	Statistical model	No. of analyses
Adkins et al. 2010		Malawi		LED lanterns (charged by solar panel)		Probit		1
Amacher et al. 1992		Nepal		Biomass ICSs		Probit		1
Amacher et al. 1996		Nepal		Biomass ICSs		Probit		2
Arthur et al. 2010		Mozambique		Fuel choice (charcoal, kerosene, electricity)		Logit		4
Chaudhuri and Pfaff 2003		Pakistan		Fuel choice (modern fuels, traditional fuels)		Engel curves, probit		1
Damte and Koch 2011		Ethiopia		Lakech ICS, Mirt ICS		Weibull regression model, exponential, Koch		2
Edwards and Langpap 2005		Guatemala		Gas ICSs		Full information maximum likelihood		2
El Tayeb Muneer and Mukhtar Mohamed 2003		Sudan		ICSs		Linear regression		1
Farsi et al. 2007		India		Fuel choice		Ordered probit		1
Gebreegziabher et al. 2009		Ethiopia		Fuel choice (wood, charcoal, kerosene, electricity), electric ICS		Probit		5
Gundimeda and Köhlin 2008		India		Fuel choice (fuelwood, kerosene, LPG, electricity)		Linear approximate almost ideal demand system		24
Gupta and Köhlin 2006		India		Fuel choice (fuelwood, coal, kerosene, LPG)		Probit		4
Heltberg 2004		Brazil, South Africa, Vietnam, Guatemala, Ghana, Nepal, and India		Fuel switching (partial to full use of nonsolid fuel, partial use of nonsolid fuel to only solid fuel)		Logit		28
Heltberg 2005		Guatemala		Fuel choice (fuelwood, LPG)		Multinomial logit		3
Hosier and Dowd 1987		Zimbabwe		Fuel choice [transitional fuels (coal and dung), fuelwood, kerosene, electricity]		Logit		10
Jack 2006		Peru		Fuel choice (wood only, gas only, wood and gas)		Probit		3
Kavi Kumar and Viswanathan 2007		India		Fuel choice [“dirty” fuel (firewood, dung, coal, and coke) vs. “clean” fuel (kerosene, gobar gas, LPG)]		Probit		12
Kebede et al. 2002		Ethiopia		Fuel choice (kerosene, butane gas, electricity)		Regression		1
Kemmler 2007		India		Fuel choice (electricity)		Probit		1
Khandker et al. 2010		India		Fuel choice (biomass, kerosene, LPG, electricity)		Tobit		8
Lamarre-Vincent 2011		Indonesia		Fuel choice (kerosene, LPG)		No fixed effects, fixed effects		1
Louw et al. 2008		South Africa		Fuel choice (electricity)		Logarithmic regression		1
McEachern and Hanson 2008		Sri Lanka		Single household solar system adoption		Multivariate linear regression		2
Ouedraogo 2006		Burkina Faso		Fuel choice (agricultural waste, cow dung, charcoal, firewood, kerosene, LPG)		Multinomial logit		4
Peng et al. 2010		China		Fuel choice (biomass, nonbiomass)		Logit		1
Pine 2011		Mexico		Patsari ICSs		Multinomial logistic regression		1
Rao and Reddy 2007		India		Fuel choice (firewood, coal, coke, dung, charcoal, kerosene, LPG)		Multinomial logit		4
Rebane and Barham 2011		Nicaragua		Solar home system		Biprobit, probit		1
Reddy 1995		Bangalore, India		Fuel choice (firewood, charcoal, kerosene, LPG, electricity)		Multinomial logit		8
Walekhwa et al. 2009		Uganda		Fuel choice (biogas)		Binomial logistic regression		1
Wendland et al. 2011		Benin and Togo		ICSs		Probit		1
Yan 2010		China		Fuel choice (wood straw, coal, LPG, electricity)		Marginal effects of multinomial logit		6


Many studies were excluded for not using regression analysis (i.e., the study did not consider multiple determinants of adoption or fuel choice). In addition, we excluded those that used regression analysis but only considered unique variables not found in other studies, such as knowledge of how to use the ICSs (Pandey and Yadama 1992). Studies that failed to meet inclusion criteria often focused on such research questions as *a*) health outcomes related to fuel or technology adoption rather than the adoption decision itself (e.g., Campbell et al. 2003; Smith-Sivertsen et al. 2009); *b*) levels of dirty fuel consumption, without a movement to clean fuel types (e.g., Baland et al. 2010; Chen et al. 2006; Rehfuess et al. 2010); and *c*) energy shares, without a possible switch to a different fuel type (e.g., Chambwera and Folmer 2007; Kaul and Liu 1992)

*Data synthesis.* We used vote counting for *a*) ICS adoption and *b*) fuel choice. We identified broad categories of factors that influence the adoption or fuel choice decision, found several variables of interest within each category [see Supplemental Material, Table 1 (http://dx.doi.org/10.1289/ehp.1104194)], and applied the vote-counting method to each variable. That is, for each analysis included, we determine whether the variable was included in the study, and if so, whether the study found a statistically nonsignificant or significant positive or negative influence on the adoption or fuel choice. We summarized the results by calculating *a*) the percentage of studies including each variable, *b*) the percentage of studies that found a statistically significant effect for a variable out of all studies that included the variable, *c*) the percentage of studies that found a statistically significant effect out of all studies.

For studies that provided data from multiple data sets (e.g., a study that analyzed decisions of switching from solid fuel to kerosene or LPG), we analyzed and counted votes from each analysis separately. Several studies analyzed the same data with different regression models—only the main regression for each data or subsample was used for this review.

We conducted vote counting to determine the impact of diverse variables on the likelihood of adopting improved energy technology: either adopting an ICSs or moving from solid fuel (charcoal or dung, agricultural residues, coal, or fuelwood) to cleaner fuels (kerosene, LPG, electricity, or solar). However, some studies analyzed fuel choice that was a backward movement on the energy ladder (e.g., switching from kerosene to biomass). In these cases, the positive or negative sign for a significant finding was reversed to maintain consistency. Studies that considered movements from one type of solid fuel to another (e.g., dung to coal) were excluded.

In addition to separating the positive and negative associations within the statistically significant associations (significance at the 1%, 5%, or 10% level, or *p*-values ≤ 0.1), we also report results that are not significant (*p* > 0.1) within the figures for ICS adoption and fuel choice.

Throughout this article, we use the term “association” to provide information on a potentially valuable relationship across all included studies. For example, an analysis that finds income to be statistically significantly and positively associated with ICS use receives a “positive significant” vote. If most analyses found a significantly positive association between income and ICS use, the income variable is said to have a positively significant association with ICS use. However, association does not imply causality.

The studies that satisfied the inclusion criteria considered > 150 variables, which were merged to form 26 variables in three categories (for a complete list of these variables, see Supplemental Material, Table 1). For example, studies used many different variables to describe household income. To represent the “income” variable in this review, we used the following proxies: income, expenditure, area of land under household management, wealth (including assets), profit from household production, income per capita, expenditure per capita, and categorization as high income.

The final variables are composed of combinations of discrete and continuous variables. Where necessary, the positive or negative vote was reversed to preserve consistency in direction of effect (e.g., the vote for the continuous variable “number of years of education in household” was treated equivalent to the vote from the discrete variable “highest education in household was secondary education,” and the reverse-sign vote of “head of household illiterate”). No analyses were double counted.

## Results

**Figure 2:**
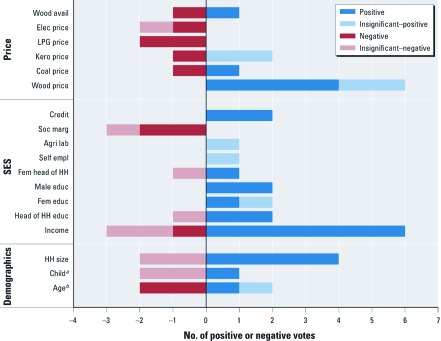
Systematic review of variables that influence the adoption of ICSs. Each analysis of ICS adoption casts one “vote” for every variable that it includes. The sign of the vote (positive or negative) reflects the direction of the association with ICS adoption. Abbreviations: agri, agriculture; avail, availability; educ, education; elec, electricity; empl, employment; kero, kerosene; lab, labor; soc marg, socially marginal status; fem, female; HH, household. ***^a^***Child is a variable created by merging three variables: presence of children in household, number of children, and proportion of children < 15 years of age. ***^b^***Age is a variable created by merging four variables: age of head of household, age of head of household if > 30 years of age, wife’s age, and mean household age (see Supplemental Material, Table 1).

**Figure 3:**
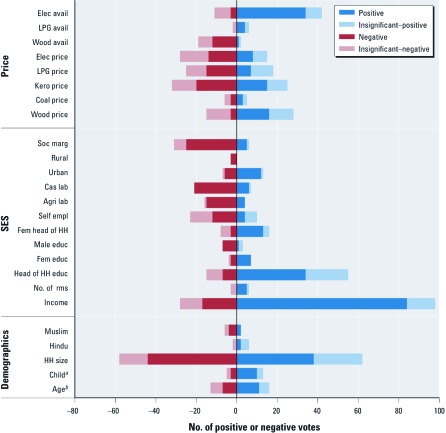
Systematic review of variables that influence choice of cooking fuels. Each analysis of clean fuel choice casts one “vote” for every variable that it includes. The sign of the vote (positive or negative) reflects the direction of the association with clean fuel choice. Abbreviations: agri, agriculture; avail, availability; cas, caual; educ, education; elec, electricity; empl, employment; kero, kerosene; lab, labor; soc marg, socially marginal status; fem, female; HH, household; rms, rooms per household. ***^a^***Child is a variable created by merging three variables: presence of children in household, number of children, and proportion of children < 15 years of age. ***^b^***Age is a variable created by merging four variables: age of head of household, age of head of household if > 30 years of age, wife’s age, and mean household age (see Supplemental Material, Table 1).

We conducted two vote counting exercises: ICS adoption (Figure 2) and clean fuel choice (Figure 3). Overall, we found that ICS adoption and clean fuel choice were significantly associated with socioeconomic status: income and education were positively associated, and socially marginalized status was negatively related. Although the meta-sample size of ICS studies that considered credit was small (*n* = 2), access to credit was positively associated with ICS adoption. We also observed a difference in clean fuel adoption by location: clean fuels were more likely to be chosen in urban areas than in rural areas.

The studies included in our analysis (Table 1) considered between 2 and 33 variables, with most most of the analyses including between 7 and 15 variables. Of the studies analyzed, 60% were from Asia (the vast majority from India), 27% from Africa, and the remainder from Latin America (13%) and 45% considered urban households, 40% considered rural households, and the remaining 15% compared urban and rural households. The sample size of the analyses ranged from 68 to 71,074 households (mean = 1,082 for ICSs, 16,810 for fuel choice; median = 300 for ICSs, 4,400 for fuel choice). Studies with large sample sizes used national household surveys and conducted multiple regression analyses. Three such studies contained 64 analyses, and we conducted a robustness check (described below) to determine whether these studies biased our results. A second robustness check eliminated all analyses from India (*n* = 67), which accounted for 46% of all the analyses. A third robustness check considered the sum of statistically significant and nonsignificant positive and negative votes.

*Improved cookstoves.* Eleven of the 146 analyses that met inclusion criteria considered the adoption of ICSs. These analyses were from eight papers and considered either the decision to purchase an ICS or the actual use of the stove [for a list of all analyses, see Supplemental Material, Table 2 (http://dx.doi.org/10.1289/ehp.1104194)]. The ICSs in these analyses included different stove models that required different types of fuel: biomass (three analyses), charcoal (one analysis), LPG (two analyses), electricity (one analysis), and an unspecified fuel (four analyses).

The ICS analyses considered 18 variables as determinants of adoption. Only three variables—household size, income, and fuelwood price—were considered by more than half of the analyses, which suggests a lack of united evaluation criteria in ICS adoption studies.

The vote-counting exercise showed that education variables have a statistically significant positive association with ICS adoption in most studies that considered this variable [head of household education, 67%; male education, 100%; for detailed vote-counting results, see Supplemental Material, Table 3 (http://dx.doi.org/10.1289/ehp.1104194)]. Two analyses considered female education; of these, one found a significantly positive association with ICS adoption. Other variables with significant positive associations with ICS adoption were income (in 67% of the analyses that considered income), fuelwood price (67%), household size (67%), and credit access (100%). The studies found a negative association between ICS adoption and age of the head of household (50%) and socially marginal status (67%), such as the scheduled caste in India [for a full list of marginal status, see Supplemental Material, Table 1). Only one study, which included two analyses, considered LPG price and found the expected result that higher LPG prices were negatively associated with the adoption of ICSs (in this case, an LPG stove). Vote counting was inconclusive for female head of household, fuelwood availability, coal price, and electricity price on rates of ICS adoption; that is, neither positive, negative, nor statistically nonsignificant findings accounted for at least 50% of votes. We also observed that most analyses found nonsignificant associations between ICS adoption and occupation (self-employment or agricultural labor), number of children, and the price of kerosene.

*Fuel choice.* Separately, we considered 135 analyses from 25 articles that examined fuel switching from solid fuel (dung, agricultural residue, biomass, charcoal, or coal) to a cleaner fuel [for a list of all analyses, see Supplemental Material, Table 4). All variables of interest except access to credit were included in at least one of the fuel choice analyses.

Only three variables—head of household education, income, and household size—were considered by more than half of the analyses, which again illustrates the lack of unified evaluation criteria (for detailed vote counting results, see Supplemental Material, Table 5). Income was included as a variable in 93% of the analyses, and most (67%) found a positive relationship with cleaner fuel use.

Demographic variables had very mixed results. Household size was considered in a high percentage of studies (89%) but was inconclusive, as was age of head of household. Households with a female head of household were more likely to use cleaner fuel (54% of studies found positive significance). Three studies (with eight total analyses) considered religion as a driver of fuel choice. Two studies from India found an nonsignificant relationship between Hindu households and the adoption of clean fuels. One study from India and one from Burkina Faso included an indicator for Muslim households: 75% of the analyses from India found a negative association with adoption, which may reflect the lower socioeconomic status of Muslims in India; 50% of the analyses from Burkina Faso showed a significantly positive relationship.

Of the three education variables considered, only female education had a positive association with cleaner fuel use (64% positive votes). Head of household education was considered in 52% of analyses but was inconclusive. Male education had a significant negative relationship with cleaner fuel use in 70% of the analyses but was considered in fewer analyses (a total of 10) than was head of household education (a total of 70). All of the analyses with a finding of statistical significance and negative association for male education are from one study in India (Khandker et al. 2010).

Households working in agricultural or casual labor, signaling their socioeconomic status, were less likely to use cleaner fuel, although self-employment was nonsignificant for fuel choice. Socially marginal status was negatively associated with use of cleaner fuels in 68% of studies that considered status. Households located in urban areas were much more likely to adopt clean fuels compared with similar households in rural areas.

Some studies directly considered fuel availability on fuel choice: good availability of electricity was positively associated with clean energy use, whereas fuelwood availability and LPG access were inconclusive. The price of all fuel options other than fuelwood was inconclusive; fuelwood price was nonsignificant. Vote counting results are shown in detail in Supplemental Material, Table 5 (http://dx.doi.org/10.1289/ehp.1104194).

*Robustness checks.* A few of the papers that matched inclusion criteria contained a very large number of regression analyses. Because the analyses in each paper considered the same variables, the papers that contained more than 12 regression analyses were removed from the analysis of fuel choice to assess the magnitude of influence they exerted on overall results and to address the risk of bias in individual studies. Of the 135 analyses that included fuel choice, 64 (47%) were from three papers (Gundimeda and Köhlin 2008; Heltberg 2004; Kavi Kumar and Viswanathan 2007) that used extensive regression analyses based on national surveys. The remaining 71 analyses (from 22 papers) were considered separately. These analyses have very similar levels of significance for the variables of interest compared with the full sample. However, three differences emerge in substitute prices: *a*) wood price is inconclusively associated with cleaner fuel use (it was nonsignificant for the complete set of fuel choice studies); *b*) LPG price is negatively associated with cleaner fuel use (inconclusive in the full set); *c*) electricity price is nonsignificant [inconclusive in full set of analyses (135)].

**Figure 4:**
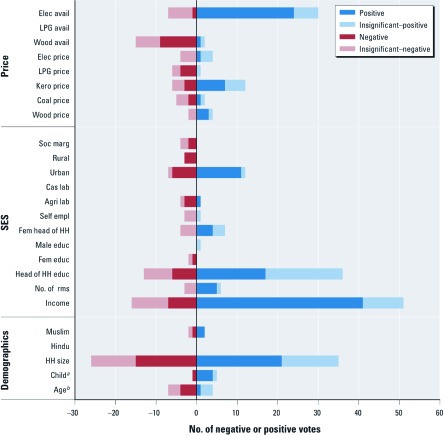
Systematic review of variables that influence choice of cooking fuels: robustness check excluding the large number of Indian analyses. Each analysis of clean fuel choice casts one “vote” for every variable that it includes. The sign of the vote (positive or negative) reflects the direction of the association with clean fuel choice. Only analyses that were conducted in countries other than India are included in this test for robustness. Abbreviations: agri, agriculture; avail, availability; cas, caual; educ, education; elec, electricity; empl, employment; kero, kerosene; lab, labor; soc marg, socially marginal status; fem, female; HH, household; rms, rooms per household. ***^a^***Child is a variable created by merging three variables: presence of children in household, number of children, and proportion of children < 15 years of age. ***^b^***Age is a variable created by merging four variables: age of head of household, age of head of household if > 30 years of age, wife’s age, and mean household age (see Supplemental Material, Table 1).

A second robustness check was conducted on the impact of a single country with a large number of analyses. A large number of the analyses (*n* = 67; 46% of all 146 analyses; 50% of the 135 fuel choice analyses) in this systematic review took place in India, all of which consider fuel choice, not ICS adoption. Therefore, eliminating the analyses of Indian fuel choice had several impacts on the results (Figure 4). All education variables were either nonsignificant (head of household and male education) or inconclusive (female education). In addition, female head of household and age of head of household were nonsignificant. Socially marginal status became nonsignificant. Muslim households switched from a negative association to a positive association, because all of the non-Indian analyses that considered the Muslim religion as a covariate were in Burkina Faso, where > 50% of the country is Muslim. Associations with prices remained a mix of inconclusive (wood and kerosene) and nonsignificant (coal and electricity) results. Only LPG price showed a significant (negative) association.

The third robustness check considered votes that were either statistically significant or nonsignificant, grouping only by the direction of the effect. For ICSs, this check indicated that all education variables had a positive association, wood and kerosene prices were positively associated, and other substitute prices (electricity and LPG) were negatively associated. For fuel choice, four inconclusive price variables (coal, kerosene, LPG, electricity) showed negative associations with clean fuel choice (as expected) when nonsignificant votes are included.

## Discussion

We present a systematic review of the current literature on stove and fuel choice of developing country households. Our evaluation considered studies that examined a step “up the energy ladder” either by *a*) adopting an ICS or *b*) choosing cleaner fuel (kerosene, LPG, electricity, or solar) over solid fuel (fuelwood, agricultural residues, coal, charcoal, or dung). We included studies that conducted statistical analyses of several potential drivers that ranged from income to demographics to location.

The studies are primarily concentrated in Asia (particularly India), with scattered research in Africa and Latin America. Most analyses were in regions with the highest burden of disease from solid fuel use (Smith et al. 2004). Additionally, 90% were in BC hotspots (areas that experience regional BC-induced atmospheric solar heating) (Ramanathan and Carmichael 2008; Ramanathan et al. 2007).

Income is the most widely studied determinant. Although inconclusive in a few studies, most studies find that households with greater income are more likely to use more expensive (and cleaner and healthier) energy. The relationship between income and adoption is influenced by the social structure; for example, in some patriarchal societies, women receive only a portion of family earnings with which to purchase fuel or an improved stove (El Tayeb Muneer and Mukhtar Mohamed 2003). Thus, we considered the sex of the head of household and found that female-headed households were more likely to adopt cleaner fuels (the ICS studies rarely included this variable). We also found that households in marginalized groups (low caste, indigenous, or regional ethnic group) were less likely to adopt clean fuel and ICSs. This relationship could be due to traditional cooking techniques that have a particular intrinsic value in certain ethnic groups or could represent an association between social marginalization and adoption.

The occupation variables offered a mixed picture—self-employment or casual labor was nonsignificant for ICS adoption, but casual and agricultural labor were negatively associated with clean fuel adoption (as expected), potentially because of lower income. Education was positively associated with ICS adoption—although more analyses found the association for head of household and for men (both more likely to oversee family expenditures) but not for women. Head of household education and female education were positively associated with cleaner fuel adoption, whereas male education was negatively associated.

Urban households were more likely to use cleaner fuels, whereas rural households were significantly less likely, as expected (DeFries and Pandey 2010). These relationships are likely due to the limited availability of clean fuels in rural settings. Access to credit was positively associated with ICS adoption.

Associations with price were varied. For example, although higher prices for dirty fuels such as wood and coal appeared to encourage adoption of ICSs, the association with fuel choice was statistically nonsignificant for fuelwood or inconclusive for coal. Again, the higher price of only LPG but not other cleaner fuels appeared to decrease the adoption of ICSs. All clean fuel prices had an inconclusive association with fuel choice. However, one of the robustness tests (excluding studies with many regressions) suggests that fuelwood and LPG price do influence fuel choices.

Systematic reviews differ from other reviews because they are based on clear inclusion and exclusion criteria and use a conceptual framework for quantification. Yet, judgment is exercised at every step of the review in order to use the evidence derived from evaluative research (Chalmers 2005; Sorrell 2007; van der Knaap et al. 2008). Thus, several caveats are necessary.

First, systematic reviews can reduce but not remove subjectivity because the technique brings together a number of studies, and the analyst is instrumental in their selection. Second, systematic reviews condense details through the process of aggregation, which glosses over any differences in research designs and measurement protocols. This arises partly because the empirical methods in most of the articles that we reviewed here were typically ecological, not experimental, studies. Unlike strict experiments, the reporting of assumptions, error distributions, and data idiosyncrasies is not standardized.

Third, publication biases persist. Although one-quarter (*n* = 8) of the total articles included in this review are unpublished reports, our search strategy may have omitted others. Fourth, the review and the scope of possible conclusions are limited by the questions, methods, and samples considered by the authors of the primary studies: the existing body of empirical research may have applied an inappropriate methodology (e.g., unaccounted for confounders) or overlooked some factors. Omission of variables should not be viewed as an implication of their irrelevance (Pattanayak et al. 2003); for example, the impact of income on ICS adoption may be misunderstood if access to credit is not considered.

Fifth, although systematic reviews can employ more rigorous techniques to derive cross-study meta-measures (e.g., a price elasticity of demand), we applied simple vote-counting methods. We cannot conduct more advanced meta-analyses (e.g., meta-regressions of effects size) or provide meta-estimates of the size of the influence for at least two reasons (Beach et al. 2005). First, unlike most meta-regressions that focus on a single effect size, we considered a multitude of associations with adoption (and related effect sizes), because we are examining the broad relationship between various factors and the propensity of adoption. Second, the authors of the primary studies used different discrete choice models and did not provide standardized marginal effects with respect to changes in the explanatory variables.

Finally, we note that adoption is a proxy—a necessary but insufficient surrogate—for the kind of behavior change that can deliver health, environmental, and climate benefits. Knowledge, attitude, and practice (adoption) go hand in hand. Thus, it is critical to obtain objective measures of proper stove use, as well as more details on users’ understanding of the emissions–health link and of the instructions for proper stove use and awareness of exposure reducing behaviors.

## Conclusion

Using a systematic review, we analyzed results from 146 analyses of studies from 32 papers conducted in 22 countries. We found evidence of a systematic and theoretically consistent relationship between adoption of clean energy products and socioeconomic status (including income, education, and social marginalization) and urban location. Overall, evidence of a positive influence of education and location suggests that strengthening the information and communication aspects of social marketing and extending the supply-chain into rural areas could increase adoption. We also found several nuances, such as the varying associations with male education (positive for ICSs, negative for clean fuel), an inconclusive result on price of substitutes (LPG, electricity), and the limited study of credit access. Different types of ICSs use different types of fuel, and the price of kerosene and LPG may not necessarily reduce the likelihood of purchasing an ICS that burns wood. When considered, credit access is strongly associated with ICS adoption, suggesting that microfinance interventions could boost the effectiveness of ICS programs.

Several variables were not widely included and were therefore excluded from our review. For example, information about ICSs or the health impacts of dirty fuels was included in only two studies (El Tayeb Muneer and Mukhtar Mohamed 2003; Gupta and Köhlin 2006). Other understudied variables included proximity to markets or salespersons (e.g., Adkins et al. 2010; Kaul and Liu 1992) and peer effects and social capital (e.g., McEachern and Hanson 2008; Saha S, Pattanayak SK, unpublished observations). More critically, potentially influential institutional variables such as democratic governance (Wendland et al. 2011) and participation in village organizations (Saha S, Pattanayak SK, unpublished observations) were simply not considered in most primary analyses.

In summary, the literature on adoption of clean energy sources by households in developing countries remains scattered and largely qualitative. Although many efforts have sought to review ICS or fuel choice, rigorous statistical confirmation is rare. In this article, we explore how this ICS–fuel choice literature provides important feedback for ongoing international efforts to disseminate millions of ICSs. Although we take a first step in summarizing the “sign” of the effect, we do not provide guidance on the “size” of the effects. Future reviews could consider employing rigorous statistical analysis to conduct meta-regressions of effect sizes of variables that have been most frequently studied (e.g., income, education).

It is beyond the scope of our review to comment on true sustained adoption of a new technology (ICSs) or cleaner fuel because none of the primary studies measure use of the ICSs or clean fuel over time. Thus, our review of clean energy adoption has mixed implications for implementation programs in developing countries. The basic theory of technology adoption suggests that household (e.g., income, attitudes) and institutional (e.g., information campaigns, supply chain) factors determine household choice (Pattanayak and Pfaff 2009). However, much of the existing quantitative research examines only a few factors such as income, education, and family size, which in turn are rough-and-ready proxies (i.e., crude estimates that are easily captured) for the complex process of technology adoption. Many relationships are still unclear, such as the relative importance of family size and composition, employment, fuelwood availability, and, most important, the cost of energy alternatives. Furthermore, we need deeper examinations of various aspects of the complex social system (e.g., intrahousehold bargaining and gender politics) and direct interventions such as health promotion, social marketing and supplier training.

Although the evidence base for individual level “demand drivers” (e.g., peer pressure) is extremely thin and not robust to settings and experiences, the pendulum appears to have swung too far from institutional level “supply drivers” (e.g., social marketing). We contend that there is a need to consider an updated theory of diffusion and adoption in which household drivers such as income, information, and attitudes are modified by *a*) underlying sociopsychological drivers (e.g., discount rates, risk aversion, conformity, peer pressure), *b*) specific programs and policies such as kerosene and LPG subsidies, *c*) product commercialization such as social marketing, cheap credit, alternative fuels, supply-chain strengthening, and *d*) the capacity and interest of local officials and nongovernmental organizations. Unfortunately, the evidence base for the combined effect of these factors is virtually nonexistent. Thus, we hope that future researchers and implementers can use our review as a stepping-stone toward learning about ICS adoption and fuel choice in order to expand what we know and what we do not know and to elucidate the many opportunities for future study.

## Supplemental Material

(229 KB) PDF
