# Supplementary material for: Who Adopts Improved Fuels and Cookstoves? A Systematic Review
Source: Environ Health Perspect. 2012 Feb 1;120(5):637–45. doi: 10.1289/ehp.1104194 (PMC3346782; doi:10.1289/ehp.1104194)
Supplement: (229 KB) PDF [file ehp.1104194.s001.pdf]

Supplemental Material

Who Adopts Improved Fuels and Cookstoves? A Systematic Review

Jessica J. Lewis[1], Subhrendu K. Pattanayak[1,2,3*]

[*]Corresponding author: Duke University, PO Box 90312, Durham, NC 27708-0312. Tel. 919-613-9306; Fax 919-684-9940. Email address: subhrendu.pattanayak@duke.edu

[1]Nicholas School of the Environment, Duke University, Durham, NC 27708

[2]Sanford School of Public Policy, Duke University, Durham, NC, 27708

[3]Global Health Institute, Duke University, Durham, NC 27708

Table of Contents

Search Strategy Employed to Search the ScienceDirect Database: 2
Table 1. Variables Merged for Systematic Review 3
Table 2. Improved Cookstove Analyses 5
Table 3. Results of Vote-Counting for Improved Cookstove Analyses 6
Table 4. Fuel Choice Analyses 7
Table 5. Results for Fuel Choice Analyses 15

Search Strategy Employed to Search the ScienceDirect Database:

- "cookstove" AND "adoption" AND "regression" in all fields
- Dissemination and regression and cookstove – all fields
- "fuel choice"(title, keywords, abstract) AND cooking AND regression (all fields)
- "fuel choice"(title, keywords, abstract) AND biomass AND regression (all fields)
- Household AND energy AND fuel AND choice OR switch OR switching (title, keywords, abstract) AND regression (all fields)
- Residential AND energy AND fuel AND choice OR switch OR switching (title, keywords, abstract) AND regression (all fields)
- domestic AND energy AND fuel AND choice OR switch OR switching (title, keywords, abstract) AND regression (all fields)
- "fuel switching" (title, keywords, abstract) and regression (all fields)
- "energy ladder" (title, keywords, abstract) and regression (all fields)
- Improved cookstove (title, keywords, abstract) and adoption and regression (all fields)
- Charcoal (title, abstract, keywords) and household and regression (all fields)
- solar and energy and adoption or switch or switching or choice or choose (title, abstract, keywords) AND household or domestic or residential AND regression (all fields)
- photovoltaic and energy and adoption or switch or switching or choice or choose (title, abstract, keywords) AND household or domestic or residential AND regression (all fields)
- electricity and adoption or switch or switching or choice or choose (title, abstract, keywords) AND household or domestic or residential AND regression (all fields)
- biogas and adoption or switch or switching or choice or choose (title, abstract, keywords) AND household or domestic or residential AND regression (all fields)
- biogas and adoption (title, abstract, keywords) AND regression (all fields)
- fuel and adoption or switch or switching or choice or choose (title, abstract, keywords) AND household or domestic or residential AND regression (all fields)
- energy and adoption or switch or switching or choice or choose (title, abstract, keywords) AND household or domestic or residential AND regression (all fields)
- cookstove and adoption or switch or switching or choice or choose (title, abstract, keywords) AND household or domestic or residential AND regression (all fields)
- biomass and adoption or switch or switching or choice or choose (title, abstract, keywords) AND household or domestic or residential AND regression (all fields)
- fuelwood and adoption or switch or switching or choice or choose (title, abstract, keywords) AND household or domestic or residential AND regression (all fields)

Supplemental Material, Table 1. Variables Merged for Systematic Review

Final Variable of Interest	Variables from included studies merged to form final variable in systematic review
Demographics	
Age	Age of head of HH<br>Age of head of HH, if >30<br>Wife's age<br>Mean household age
Children	Presence of children in HH (yes)<br># children<br>Proportion of children under 15
Household Size	HH size<br>HH size >=10
Hindu	Hindu<br>Non-Hindu*
Muslim	Muslim
Socio-Economic Status (SES)	
Income	Income<br>Expenditure<br>Land under household management (proxy for income)<br>Wealth (including assets)<br>Profit from household production<br>Income per capita<br>Expenditure per capita<br>High income category<br>Electric goods (both electricity connection and ownership of electric appliances)
Number of Rooms in House	Number of rooms in house
Head of Household Education	Higher Education of Head of HH<br>Education of Head of HH (years),<br>Head of HH secondary education<br>Head of HH primary education<br># of people in household with education (primary and higher)<br>Max education in HH is secondary<br># years of education of everyone in household<br>Max education in household (# years)<br>Head of HH Illiterate*
Female Education	# of years of female head of HH’s education<br>Wife's educational level<br>Wife Illiterate*<br>Wife secondary or higher education
Male Education	Husband’s education, primary<br>Education of respondent's husband/father<br># years education of male head of HH<br>Husband illiterate*

Supplemental Material, Table 1. Variables Merged for Systematic Review (Continued)

Final Variable of Interest	Variables from included studies merged to form final variable in systematic review
Gender of Head of Household	Female head of HH<br>Male head of HH*
Self Employed	Self Employed
Agricultural Laborer	Agricultural Laborer<br>Farming household<br>"Does HH earn income from cotton?"
Casual Laborer	Casual Laborer
Rural	Rural
Urban	Urban
Socially Mariginalized	Forward Caste*<br>Scheduled Caste/Tribe<br>Lower Caste Dummy<br>Ethnic Group<br>Indigenous
Access to credit	Access to credit
Price	
Wood Price	Wood price
Coal Price	Coal price
Kerosene Price	Market price of kerosene<br>Ratio of kerosene to electricity price<br>Kerosene expenditure
LPG Price	LPG Price
Electricity Price	Price of electricity
Wood Availability	Availability of wood is good<br>Community median distance to firewood<br>Forest in the area<br>Distance from fuelwood entry to town<br>Distance to Forest
LPG Availability	Availability of LPG is good
Electricity Availability	Electricity in home<br>Village electrified<br>Electricity available

*Denotes a reverse-merge, in which direction of effect was reversed to preserve consistency in direction of effect

Supplemental Material, Table 2. Improved Cookstove Analyses

Author (s)	Year of Pub.	Study	Country	Type of Cleaner Technology (Stove Fuel)	Statistical Model	Sample size (HH)	# Covariates
**Amacher et al.**	1992	The adoption of consumption technologies under uncertainty: a case of improved stoves in Nepal	Nepal	Improved Cookstove (Unspecified)	Probit	99	6
**Amacher et al.**	1996	Household fuelwood demand and supply in Nepal's Tarai and Mid-Hills: Choice between cash outlays and labor opportunity	Nepal: Tarai (Gangetic Plain)	Improved Cookstove (Unspecified)	Probit	286	13
**Amacher et al.**	1996	Household fuelwood demand and supply in Nepal's Tarai and Mid-Hills: Choice between cash outlays and labor opportunity	Nepal: Mid-Hills	Improved Cookstove (Unspecified)	Probit	240	12
**Damte and Koch**	2011	Clean Fuel Saving Technology Adoption in Urban Ethiopia	Ethiopia	Mirt Improved Cookstove (Charcoal)	Weibull Regression Model	1557	15
**Damte and Koch**	2011	Clean Fuel Saving Technology Adoption in Urban Ethiopia	Ethiopia	Lakech Improved Cookstove (Biomass)	Weibull Regression Model	1557	15
**Edwards & Langpap**	2005	Startup Costs and the Decision to Switch from Firewood to Gas Fuel	Guatemala (Urban Sample)	Improved Cookstove (Gas)	Full Information Maximum Likelihood	3,424	8
**Edwards & Langpap**	2005	Startup Costs and the Decision to Switch from Firewood to Gas Fuel	Guatemala (Rural Sample)	Improved Cookstove (Gas)	Full Information Maximum Likelihood	3,852	8
**El Tayeb Muneer & Mukhtar Mohamed**	2003	Adoption of biomass improved cookstoves in a patriarchal society: an example from Sudan	Sudan	Improved Cookstove (Biomass)	Linear Regression	300	10
**Gebreegziabher et al.**	2009	Urban Energy Transition and Technology Adoption: The case of Tigrai, Northern Ethiopia	Ethiopia	Improved Mitad Cookstoves (Electric)	Probit	350	8
**Pine**	2011	Adoption and use of improved biomass stoves in Rural Mexico	Mexico	Improved Patsari Cookstove (Biomass)	Multinomial logistic regression	101	11
**Wendland et al.**	2011	Democracy and Dictatorship: Comparing household innovation across the border of Benin and Togo	Benin and Togo	Improved Cookstove (Unspecified)	Probit	135	11

Supplemental Material, Table 3. Results of Vote-Counting for Improved Cookstove Analyses (n=11)

Category	Demographics			SES									Price					
**Variable**	Age	Children	HH Size	Income	HH Educ.	Fem. Educ.	Male Educ.	Female HH	Self Empl.	Agri. Lab.	Soc. Marg.	Credit Acc.	Wood Price	Coal Price	Kero. Price	LPG Price	Elec. Price	Wood Avail.
Included	4	3	6	9	3	2	2	2	1	1	3	2	6	2	3	2	2	2
Included %	36	27	55	82	27	18	18	18	9	9	27	18	55	18	27	18	18	18
**Positive Signif. %**	25	33	67	67	67	50	100	50	0	0	0	100	67	50	0	0	0	50
**Positive Insignif. %**	25	0	0	0	0	50	0	0	100	100	0	0	33	0	67	0	0	0
**Positive Total %**	**50**	**33**	**67**	**67**	**67**	**100**	**100**	**50**	**100**	**100**	**0**	**100**	**100**	**50**	**67**	**0**	**0**	**50**
**Negative Signif. %**	50	0	0	11	0	0	0	0	0	0	67	0	0	50	33	100	50	50
**Negative Insignif. %**	0	67	33	22	33	0	0	50	0	0	33	0	0	0	0	0	50	0
**Negative Total %**	**50**	**67**	**33**	**33**	**33**	**0**	**0**	**50**	**0**	**0**	**100**	**0**	**0**	**50**	**33**	**100**	**100**	**50**
Signif. % (included studies)	75	33	67	78	67	50	100	50	0	0	67	100	67	100	33	100	50	100
Signif. % (all studies)	27	9	36	64	18	9	18	9	0	0	18	18	36	18	9	18	9	18

Positive and negative percentages are calculated as (number of votes)/(number of studies including the variable).

Abbreviations: HH Educ.= Household Education; Fem Educ.= Female Education; Male Educ.= Male Education; Female HH= Female Head of Household; Soc. Marg.= Socially Marginalized Group; Self Empl.=Self Employed; Agri. Lab.=Agricultural Laborer; Credit Acc.= Access to Credit; Kero.Price= Price of Kerosene; Elec. Price=Price of Electricity; Wood Avail.=Wood Availability

Supplemental Material, Table 4. Fuel Choice Analyses (n=135)

Author (s)	Date of Pub.	Study	Country	Fuel Choice/ Type of Cleaner Technology	Stat. Model	Sample size (HH)	# Var
Adkins et al.	2010	Off-grid energy services for the poor: Introducing LED lighting in the Millennium Villages Project in Malawi	Malawi	LED lanterns charged by solar panel	Probit	68	7
Arthur et al.	2010	On the adoption of electricity as a domestic source by Mozambican households	Mozambique	Fuel Choice: Odds of being a Charcoal consumer	Logistic regression	8377	10
Arthur et al.	2010	On the adoption of electricity as a domestic source by Mozambican households	Mozambique	Fuel Choice: Odds of being a kerosene consumer	Logistic regression	8377	10
Arthur et al.	2010	On the adoption of electricity as a domestic source by Mozambican households	Mozambique	Fuel Choice: Odds of being an electricity consumer	Logistic regression	8377	10
Arthur et al.	2010	On the adoption of electricity as a domestic source by Mozambican households	Mozambique	Fuel Choice: Odds of being an electricity consumer	Logistic regression	8377	12
Chaudhuri and Pfaff	2003	Fuel-choice and indoor air quality: a household-level perspective on economic growth and the environment	Pakistan: Urban and Rural	Fuel choice to Modern Fuels: Natural gas, LPG, kerosene	Probit	4106	5
Farsi et al.	2007	Fuel choices in Urban Indian Households	India	Fuel Choices (alternative in order: firewood, kerosene, LPG)	Ordered Probit	41,593	17
Gebreegziabher et al.	2009	Urban Energy Transition and Technology Adoption: The case of Tigrai, Northern Ethiopia	Ethiopia	Fuel Choices: Wood	Probit	350	9
Gebreegziabher et al.	2009	Urban Energy Transition and Technology Adoption: The case of Tigrai, Northern Ethiopia	Ethiopia	Fuel Choices: Charcoal	Probit	350	9
Gebreegziabher et al.	2009	Urban Energy Transition and Technology Adoption: The case of Tigrai, Northern Ethiopia	Ethiopia	Fuel Choices: Kerosene	Probit	350	9
Gebreegziabher et al.	2009	Urban Energy Transition and Technology Adoption: The case of Tigrai, Northern Ethiopia	Ethiopia	Fuel Choices: Electricity	Probit	350	9
Gundimeda & Köhlin	2008	Fuel demand elasticities for energy and environmental policies: Indian sample survey evidence	India	Fuel Choices: Fuelwood for low income rural household	Probit	12296	15
Gundimeda & Köhlin	2008	Fuel demand elasticities for energy and environmental policies: Indian sample survey evidence	India	Fuel Choices: Fuelwood for median income rural household	Probit	46923	15

Supplemental Material, Table 4. Fuel Choice Analyses (n=135) (Continued)

Author (s)	Date of Pub.	Study	Country	Fuel Choice/ Type of Cleaner Technology	Stat. Model	Sample size (HH)	# Var
Gundimeda & Köhlin	2008	Fuel demand elasticities for energy and environmental policies: Indian sample survey evidence	India	Fuel Choices: Fuelwood for high income rural household	Probit	12742	15
Gundimeda & Köhlin	2008	Fuel demand elasticities for energy and environmental policies: Indian sample survey evidence	India	Fuel Choices: Kerosene, for low income rural household	Probit	12296	15
Gundimeda & Köhlin	2008	Fuel demand elasticities for energy and environmental policies: Indian sample survey evidence	India	Fuel Choices: Kerosene, for medium income rural household	Probit	46923	15
Gundimeda & Köhlin	2008	Fuel demand elasticities for energy and environmental policies: Indian sample survey evidence	India	Fuel Choices: Kerosene, for high income rural household	Probit	12742	15
Gundimeda & Köhlin	2008	Fuel demand elasticities for energy and environmental policies: Indian sample survey evidence	India	Fuel Choices: Electricity, for low income rural household	Probit	12296	15
Gundimeda & Köhlin	2008	Fuel demand elasticities for energy and environmental policies: Indian sample survey evidence	India	Fuel Choices: Electricity, for medium income rural household	Probit	46923	15
Gundimeda & Köhlin	2008	Fuel demand elasticities for energy and environmental policies: Indian sample survey evidence	India	Fuel Choices: Electricity, for high income rural household	Probit	12742	15
Gundimeda & Köhlin	2008	Fuel demand elasticities for energy and environmental policies: Indian sample survey evidence	India	Fuel Choices: LPG, for low income rural household	Probit	12296	15
Gundimeda & Köhlin	2008	Fuel demand elasticities for energy and environmental policies: Indian sample survey evidence	India	Fuel Choices: LPG, for medium income rural household	Probit	46923	15
Gundimeda & Köhlin	2008	Fuel demand elasticities for energy and environmental policies: Indian sample survey evidence	India	Fuel Choices: LPG, for high income rural household	Probit	12742	15
Gundimeda & Köhlin	2008	Fuel demand elasticities for energy and environmental policies: Indian sample survey evidence	India	Fuel Choices: Fuelwood for low income urban household	Probit	7430	15
Gundimeda & Köhlin	2008	Fuel demand elasticities for energy and environmental policies: Indian sample survey evidence	India	Fuel Choices: Fuelwood for median income urban household	Probit	30937	15

Supplemental Material, Table 4. Fuel Choice Analyses (n=135) (Continued)

Author (s)	Date of Pub.	Study	Country	Fuel Choice/ Type of Cleaner Technology	Stat. Model	Sample size (HH)	# Var
Gundimeda & Köhlin	2008	Fuel demand elasticities for energy and environmental policies: Indian sample survey evidence	India	Fuel Choices: Fuelwood for high income urban household	Probit	8810	15
Gundimeda & Köhlin	2008	Fuel demand elasticities for energy and environmental policies: Indian sample survey evidence	India	Fuel Choices: Kerosene, for low income urban household	Probit	7430	15
Gundimeda & Köhlin	2008	Fuel demand elasticities for energy and environmental policies: Indian sample survey evidence	India	Fuel Choices: Kerosene, for medium income urban household	Probit	30937	15
Gundimeda & Köhlin	2008	Fuel demand elasticities for energy and environmental policies: Indian sample survey evidence	India	Fuel Choices: Kerosene, for high income urban household	Probit	8810	15
Gundimeda & Köhlin	2008	Fuel demand elasticities for energy and environmental policies: Indian sample survey evidence	India	Fuel Choices: Electricity, for low income urban household	Probit	7430	15
Gundimeda & Köhlin	2008	Fuel demand elasticities for energy and environmental policies: Indian sample survey evidence	India	Fuel Choices: Electricity, for medium income urban household	Probit	30937	15
Gundimeda & Köhlin	2008	Fuel demand elasticities for energy and environmental policies: Indian sample survey evidence	India	Fuel Choices: Electricity, for high income urban household	Probit	8810	15
Gundimeda & Köhlin	2008	Fuel demand elasticities for energy and environmental policies: Indian sample survey evidence	India	Fuel Choices: LPG, for low income urban household	Probit	7430	15
Gundimeda & Köhlin	2008	Fuel demand elasticities for energy and environmental policies: Indian sample survey evidence	India	Fuel Choices: LPG, for medium income urban household	Probit	30937	15
Gundimeda & Köhlin	2008	Fuel demand elasticities for energy and environmental policies: Indian sample survey evidence	India	Fuel Choices: LPG, for high income urban household	Probit	8810	15
Gupta & Köhlin	2006	Preferences for domestic fuel: Analysis with socio-economic factors and rankings in Kolkata, India	India	Fuel Choice: Fuelwood	Probit	500	16
Gupta & Köhlin	2006	Preferences for domestic fuel: Analysis with socio-economic factors and rankings in Kolkata, India	India	Fuel Choice: Coal	Probit	500	16

Supplemental Material, Table 4. Fuel Choice Analyses (n=135) (Continued)

Author (s)	Date of Pub.	Study	Country	Fuel Choice/ Type of Cleaner Technology	Stat. Model	Sample size (HH)	# Var
Gupta & Köhlin	2006	Preferences for domestic fuel: Analysis with socio-economic factors and rankings in Kolkata, India	India	Fuel Choice: Kerosene	Probit	500	16
Gupta & Köhlin	2006	Preferences for domestic fuel: Analysis with socio-economic factors and rankings in Kolkata, India	India	Fuel Choice: LPG	Probit	500	16
Heltberg	2004	Fuel Switching: Evidence from eight developing countries	Brazil - Urban	Fuel Switching from partial use of solid fuel to only using solid fuel	Logit	3,568	7
Heltberg	2004	Fuel Switching: Evidence from eight developing countries	Brazil - Urban	Fuel Switching from partial to full use of non-solid fuel	Logit	3,568	7
Heltberg	2004	Fuel Switching: Evidence from eight developing countries	South Africa - Urban	Fuel Switching from partial use of solid fuel to only using solid fuel	Logit	4,412	7
Heltberg	2004	Fuel Switching: Evidence from eight developing countries	South Africa - Urban	Fuel Switching from partial to full use of non-solid fuel	Logit	4,412	7
Heltberg	2004	Fuel Switching: Evidence from eight developing countries	Vietnam - Urban	Fuel Switching from partial use of solid fuel to only using solid fuel	Logit	1,729	7
Heltberg	2004	Fuel Switching: Evidence from eight developing countries	Vietnam - Urban	Fuel Switching from partial to full use of non-solid fuel	Logit	1,729	7
Heltberg	2004	Fuel Switching: Evidence from eight developing countries	Guatemala - Urban	Fuel Switching from partial use of solid fuel to only using solid fuel	Logit	3,387	7
Heltberg	2004	Fuel Switching: Evidence from eight developing countries	Guatemala - Urban	Fuel Switching from partial to full use of non-solid fuel	Logit	3,387	7
Heltberg	2004	Fuel Switching: Evidence from eight developing countries	Ghana - Urban	Fuel Switching from partial use of solid fuel to only using solid fuel	Logit	2,174	7
Heltberg	2004	Fuel Switching: Evidence from eight developing countries	Ghana - Urban	Fuel Switching from partial to full use of non-solid fuel	Logit	2,174	7
Heltberg	2004	Fuel Switching: Evidence from eight developing countries	Nepal - Urban	Fuel Switching from partial use of solid fuel to only using solid fuel	Logit	715	7
Heltberg	2004	Fuel Switching: Evidence from eight developing countries	Nepal - Urban	Fuel Switching from partial to full use of non-solid fuel	Logit	715	7
Heltberg	2004	Fuel Switching: Evidence from eight developing countries	India - Urban	Fuel Switching from partial use of solid fuel to only using solid fuel	Logit	46,886	7

Supplemental Material, Table 4. Fuel Choice Analyses (n=135) (Continued)

Author (s)	Date of Pub.	Study	Country	Fuel Choice/ Type of Cleaner Technology	Stat. Model	Sample size (HH)	# Var
Heltberg	2004	Fuel Switching: Evidence from eight developing countries	India - Urban	Fuel Switching from partial to full use of non-solid fuel	Logit	46,886	7
Heltberg	2004	Fuel Switching: Evidence from eight developing countries	Brazil - Rural	Fuel Switching from partial use of solid fuel to only using solid fuel	Logit	1,078	7
Heltberg	2004	Fuel Switching: Evidence from eight developing countries	Brazil - Rural	Fuel Switching from partial to full use of non-solid fuel	Logit	1,078	7
Heltberg	2004	Fuel Switching: Evidence from eight developing countries	South Africa - Rural	Fuel Switching from partial use of solid fuel to only using solid fuel	Logit	4,301	7
Heltberg	2004	Fuel Switching: Evidence from eight developing countries	South Africa - Rural	Fuel Switching from partial to full use of non-solid fuel	Logit	4,301	7
Heltberg	2004	Fuel Switching: Evidence from eight developing countries	Vietnam - Rural	Fuel Switching from partial use of solid fuel to only using solid fuel	Logit	4,269	7
Heltberg	2004	Fuel Switching: Evidence from eight developing countries	Vietnam - Rural	Fuel Switching from partial to full use of non-solid fuel	Logit	4,269	7
Heltberg	2004	Fuel Switching: Evidence from eight developing countries	Guatemala - Rural	Fuel Switching from partial use of solid fuel to only using solid fuel	Logit	3,848	7
Heltberg	2004	Fuel Switching: Evidence from eight developing countries	Guatemala - Rural	Fuel Switching from partial to full use of non-solid fuel	Logit	3,848	7
Heltberg	2004	Fuel Switching: Evidence from eight developing countries	Ghana - Rural	Fuel Switching from partial use of solid fuel to only using solid fuel	Logit	3,758	7
Heltberg	2004	Fuel Switching: Evidence from eight developing countries	Ghana - Rural	Fuel Switching from partial to full use of non-solid fuel	Logit	3,758	7
Heltberg	2004	Fuel Switching: Evidence from eight developing countries	Nepal - Rural	Fuel Switching from partial use of solid fuel to only using solid fuel	Logit	2,657	7
Heltberg	2004	Fuel Switching: Evidence from eight developing countries	Nepal - Rural	Fuel Switching from partial to full use of non-solid fuel	Logit	2,657	7
Heltberg	2004	Fuel Switching: Evidence from eight developing countries	India - Rural	Fuel Switching from partial use of solid fuel to only using solid fuel	Logit	70,474	7
Heltberg	2004	Fuel Switching: Evidence from eight developing countries	India - Rural	Fuel Switching from partial to full use of non-solid fuel	Logit	70,474	7
Heltberg	2005	Factors determining household fuel choice in Guatemala	Guatemala	Fuel Choices: Urban LPG only (relative to rural wood and LPG)	Multinomial logit	2,845	21

Supplemental Material, Table 4. Fuel Choice Analyses (n=135) (Continued)

Author (s)	Date of Pub.	Study	Country	Fuel Choice/ Type of Cleaner Technology	Stat. Model	Sample size (HH)	# Var
Heltberg	2005	Factors determining household fuel choice in Guatemala	Guatemala	Fuel Choices: Rural LPG only (relative to rural wood and LPG)	Multinomial logit	3,385	21
Heltberg	2005	Factors determining household fuel choice in Guatemala	Guatemala	Fuel Choices: Rural Wood Only (relative to rural wood and LPG)	Multinomial logit	3,385	21
Hosier and Dowd	2005	Household Fuel Choice in Zimbabwe	Zimbabwe	Fuel Choice: Gathered fuel wood to electricity	Logit	1865	10
Hosier and Dowd	1987	Household Fuel Choice in Zimbabwe	Zimbabwe	Fuel Choice: Gathered fuel wood to kerosene	Logit	1865	10
Hosier and Dowd	1987	Household Fuel Choice in Zimbabwe	Zimbabwe	Fuel Choice: gathered fuel wood to Transitional fuels (i.e., coal and dung)	Logit	1865	10
Hosier and Dowd	1987	Household Fuel Choice in Zimbabwe	Zimbabwe	Fuel Choice: Gathered fuelwood to purchased fuelwood	Logit	1865	10
Hosier and Dowd	1987	Household Fuel Choice in Zimbabwe	Zimbabwe	Fuel Choice: Kerosene to Electricity	Logit	1865	10
Hosier and Dowd	1987	Household Fuel Choice in Zimbabwe	Zimbabwe	Fuel Choice: Transitional fuels (i.e., coal and dung) to Kerosene	Logit	1865	10
Hosier and Dowd	1987	Household Fuel Choice in Zimbabwe	Zimbabwe	Fuel Choice: Purchased fuelwood to kerosene	Logit	1865	10
Hosier and Dowd	1987	Household Fuel Choice in Zimbabwe	Zimbabwe	Fuel Choice: Purchased fuelwood to transitional fuels (i.e., coal and dung)	Logit	1865	10
Hosier and Dowd	1987	Household Fuel Choice in Zimbabwe	Zimbabwe	Feul Choice: Transitional fuels (i.e., coal and dung) to Electricity	Logit	1865	10
Hosier and Dowd	1987	Household Fuel Choice in Zimbabwe	Zimbabwe	Fuel Choice: Purchased fuelwood to electricity	Logit	1865	10
Jack	2006	Household behavior and energy demand: Evidence from Peru	Peru	Wood Only	Pooled ordered probit	15922	13
Jack	2006	Household behavior and energy demand: Evidence from Peru	Peru	Wood and Gas	Pooled ordered probit	15922	13
Jack	2006	Household behavior and energy demand: Evidence from Peru	Peru	Gas Only	Pooled ordered probit	15922	13
Kavi Kumar and Viswanathan	1987	Changing structure of income indoor air pollution relationship in India	India	Fuel Choice: "Dirty" fuel (firewood, dung, coal, and coke), RURAL	Probit	71074	3
Kavi Kumar and Viswanathan	2002	Changing structure of income indoor air pollution relationship in India	India	Fuel Choice: "Dirty" fuel (firewood, dung, coal, and coke), RURAL	Probit	61696	3

Supplemental Material, Table 4. Fuel Choice Analyses (n=135) (Continued)

Author (s)	Date of Pub.	Study	Country	Fuel Choice/ Type of Cleaner Technology	Stat. Model	Sample size (HH)	# Var
Kavi Kumar and Viswanathan	2007	Changing structure of income indoor air pollution relationship in India	India	Fuel Choice: "Dirty" fuel (firewood, dung, coal, and coke), RURAL	Probit	63478	3
Kavi Kumar and Viswanathan	2007	Changing structure of income indoor air pollution relationship in India	India	Fuel Choice: "Clean" fuel (kerosene, gobar gas, LPG), RURAL	Probit	71033	3
Kavi Kumar and Viswanathan	2007	Changing structure of income indoor air pollution relationship in India	India	Fuel Choice: "Clean" fuel (kerosene, gobar gas, LPG), RURAL	Probit	61640	3
Kavi Kumar and Viswanathan	2007	Changing structure of income indoor air pollution relationship in India	India	Fuel Choice: "Clean" fuel (kerosene, gobar gas, LPG), RURAL	Probit	63307	3
Kavi Kumar and Viswanathan	2007	Changing structure of income indoor air pollution relationship in India	India	Fuel Choice: "Dirty" fuel (firewood, dung, coal, and coke), URBAN	Probit	71074	3
Kavi Kumar and Viswanathan	2007	Changing structure of income indoor air pollution relationship in India	India	Fuel Choice: "Dirty" fuel (firewood, dung, coal, and coke), URBAN	Probit	61696	3
Kavi Kumar and Viswanathan	2007	Changing structure of income indoor air pollution relationship in India	India	Fuel Choice: "Dirty" fuel (firewood, dung, coal, and coke), URBAN	Probit	63478	3
Kavi Kumar and Viswanathan	2007	Changing structure of income indoor air pollution relationship in India	India	Fuel Choice: "Clean" fuel (kerosene, gobar gas, LPG), URBAN	Probit	71033	3
Kavi Kumar and Viswanathan	2007	Changing structure of income indoor air pollution relationship in India	India	Fuel Choice: "Clean" fuel (kerosene, gobar gas, LPG), URBAN	Probit	61640	3
Kavi Kumar and Viswanathan	2007	Changing structure of income indoor air pollution relationship in India	India	Fuel Choice: "Clean" fuel (kerosene, gobar gas, LPG), URBAN	Probit	63307	3
Kebede et al.	2007	Can the urban poor afford modern energy? The case of Ethiopia	Ethiopia	Fuel Choice: Modern Fuels (Kerosene, butane gas, electricity)	Regression	4836	2
Kemmler	2007	Factors influencing houshold access to electricity in India	India	Fuel Choice: Electricity	Probit	59543	33
Khandker et al.	2010	Energy Poverty in Rural and Urban India: Are th Energy Poor Also Income Poor?	India: Rural	Biomass	Tobit	22583	12
Khandker et al.	2010	Energy Poverty in Rural and Urban India: Are th Energy Poor Also Income Poor?	India: Rural	Kerosene	Tobit	22583	12
Khandker et al.	2010	Energy Poverty in Rural and Urban India: Are th Energy Poor Also Income Poor?	India: Rural	LPG	Tobit	22583	12
Khandker et al.	2010	Energy Poverty in Rural and Urban India: Are th Energy Poor Also Income Poor?	India: Rural	Electricity	Tobit	22583	12

Supplemental Material, Table 4. Fuel Choice Analyses (n=135) (Continued)

Author (s)	Date of Pub.	Study	Country	Fuel Choice/ Type of Cleaner Technology	Stat. Model	Sample size (HH)	# Var
Khandker et al.	2010	Energy Poverty in Rural and Urban India: Are th Energy Poor Also Income Poor?	India: Urban	Biomass	Tobit	12625	12
Khandker et al.	2010	Energy Poverty in Rural and Urban India: Are th Energy Poor Also Income Poor?	India: Urban	Kerosene	Tobit	12625	12
Khandker et al.	2010	Energy Poverty in Rural and Urban India: Are th Energy Poor Also Income Poor?	India: Urban	LPG	Tobit	12625	12
Khandker et al.	2010	Energy Poverty in Rural and Urban India: Are th Energy Poor Also Income Poor?	India: Urban	Electricity	Tobit	12625	12
Lamarre-Vincent	2011	Household determinants and respiratory health impacts of fuel switching in Indonesia	Indonesia	Switching to clean fuel in 2000	No fixed effects	4698	13
Louw	2007	Determinants of electricity demand for newly electrfied low-income African households	South Africa	Fuel Choice: Electricity	Logarthmic Regression	68	7
McEachern and Hanson	2008	Socio-geographic perception in the diffusion of innovation: Solar energy technology in Sri Lanka	Sri Lanka	Single Household Solar System adoption in mature SHS adoption market villages (<=30 months since first SHS)	Multivariate linear regression	73 villages	5
McEachern and Hanson	2008	Socio-geographic perception in the diffusion of innovation: Solar energy technology in Sri Lanka	Sri Lanka	Single Household Solar System adoption in villages that newly adopted SHS (<30 months since first SHS)	Multivariate linear regression	47 villages	5
Ouedraogo	2006	Household energy preferences for cooking in urban Ouagadougou, Burkina Faso	Burkina Faso	Fuel Choices: Natural Gas	Multinomial Logit	1,008	14
Ouedraogo	2006	Household energy preferences for cooking in urban Ouagadougou, Burkina Faso	Burkina Faso	Fuel Choices: Charcoal	Multinomial Logit	1,008	14
Ouedraogo	2006	Household energy preferences for cooking in urban Ouagadougou, Burkina Faso	Burkina Faso	Fuel Choices: Firewood	Multinomial Logit	1,008	14
Ouedraogo	2006	Household energy preferences for cooking in urban Ouagadougou, Burkina Faso	Burkina Faso	Fuel Choices: Kerosene	Multinomial Logit	1,008	14
Peng	2010	Household level fuel switching in rural Hubei	China	Biomass	Logit	401	8

Supplemental Material, Table 4. Fuel Choice Analyses (n=135) (Continued)

Author (s)	Date of Pub.	Study	Country	Fuel Choice/ Type of Cleaner Technology	Stat. Model	Sample size (HH)	# Var
Rao & Reddy	2007	Variations in energy use by Indian households: An analysis of micro level data	India - rural with state dummies	Fuel Choice: LPG over Firewood	Multinomial Logit	70000	19
Rao & Reddy	2007	Variations in energy use by Indian households: An analysis of micro level data	India - rural with state dummies	Fuel Choice: Kerosene over Firewood	Multinomial Logit	70000	19
Rao & Reddy	2007	Variations in energy use by Indian households: An analysis of micro level data	India - urban with state dummies	Fuel Choice: LPG over Firewood	Multinomial Logit	48000	19
Rao & Reddy	2007	Variations in energy use by Indian households: An analysis of micro level data	India - urban with state dummies	Fuel Choice: Kerosene over Firewood	Multinomial Logit	48000	19
Rebane and Barham	2011	Knowledge and Adoption of Solar Home Systems in Rural Nicaragua	Nicaragua	Solar home system adoption	Standard Probit	158	10
Reddy	1995	A multilogit model for fuel shifts in the domestic sector	Bangalore, India	Fuel Choice: Charcoal over firewood	Multilogit	1000	9
Reddy	1995	A multilogit model for fuel shifts in the domestic sector	Bangalore, India	Fuel Choice: Kerosene over firewood	Multilogit	1000	9
Reddy	1995	A multilogit model for fuel shifts in the domestic sector	Bangalore, India	Fuel Choice: LPG over Firewood	Multilogit	1000	9
Reddy	1995	A multilogit model for fuel shifts in the domestic sector	Bangalore, India	Fuel Choice: Electricity over firewood	Multilogit	1000	9
Reddy	1995	A multilogit model for fuel shifts in the domestic sector	Bangalore, India	Fuel Choice:Kerosene over charcoal	Multilogit	1000	9
Reddy	1995	A multilogit model for fuel shifts in the domestic sector	Bangalore, India	Fuel Choice: LPG over charcoal	Multilogit	1000	9
Reddy	1995	A multilogit model for fuel shifts in the domestic sector	Bangalore, India	Fuel Choice: Electricity over charcoal	Multilogit	1000	9
Reddy	1995	A multilogit model for fuel shifts in the domestic sector	Bangalore, India	Fuel Choice: LPG over kerosene	Multilogit	1000	9
Reddy	1995	A multilogit model for fuel shifts in the domestic sector	Bangalore, India	Fuel Choice: Electricity over kerosene	Multilogit	1000	9
Walekhwa et al.	2009	Biogas energy from family-sized digesters in uganda: Critical factros and policy implications	Uganda	Fuel Choice: Biogas	Binomial Logistic Regression	220	10
Yan	2010	The Theoretical and Empirical Analysis on the Compatibility of Sustainable Development Strategies and Poverty Reduction Policies at Micro Level	China	Fuel choice: Coal over Electricity	Multinomial logit	?	18

Supplemental Material, Table 4. Fuel Choice Analyses (n=135) (Continued)

Author (s)	Date of Pub.	Study	Country	Fuel Choice/ Type of Cleaner Technology	Stat. Model	Sample size (HH)	# Var
Yan	2010	The Theoretical and Empirical Analysis on the Compatibility of Sustainable Development Strategies and Poverty Reduction Policies at MicroLevel	China	Fuel choice: LPG over Electricity	Multinomial logit	?	18
Yan	2010	The Theoretical and Empirical Analysis on the Compatibility of Sustainable Development Strategies and Poverty Reduction Policies at Micro Level	China	Fuel choice: Wood Straw over Electricity	Multinomial logit	?	18
Yan	2010	The Theoretical and Empirical Analysis on the Compatibility of Sustainable Development Strategies and Poverty Reduction Policies at Micro Level	China	Fuel choice: Coal over Electricity	Multinomial logit	4400	18
Yan	2010	The Theoretical and Empirical Analysis on the Compatibility of Sustainable Development Strategies and Poverty Reduction Policies at Micro Level	China	Fuel choice: LPG over Electricity	Multinomial logit	4400	18
Yan	2010	The Theoretical and Empirical Analysis on the Compatibility of Sustainable Development Strategies and Poverty Reduction Policies at Micro Level	China	Fuel choice: Wood Straw over Electricity	Multinomial logit	4400	18

Supplemental Material, Table 5. Results for Fuel Choice Analyses (n = 135)

Category	Demographics					Socio-Economic Status (SES)												Price							
**Variable**	Age	Child	HH Size	Hindu	Muslim	Income	# Rms	HH Educ.	Fem Educ.	Male Educ.	Fem. HH	Self Empl.	Agri. Lab.	Cas. Lab.	Urban	Rural	Soc. Marg.	Wood Price	Coal Price	Kero. Price	LPG Price	Elec. Price	Wood Avail.	LPG Avail.	Elec. Avail.
Included	29	18	120	8	8	126	9	70	11	10	24	33	20	28	20	3	37	43	11	57	43	43	21	8	53
Included %	21	13	89	6	6	93	7	52	8	7	18	24	15	21	15	2	27	32	8	42	32	32	16	6	39
Positive Signif. %	38	56	32	25	25	67	56	49	64	10	54	12	20	21	60	0	14	37	27	26	16	19	5	50	64
Positive Insignif. %	17	17	20	50	0	11	11	30	0	20	13	18	0	4	5	0	3	28	18	18	26	16	5	25	15
**Positive Total %**	55	72	52	75	25	78	67	79	64	30	67	30	20	25	65	0	16	65	45	44	42	35	10	75	79
Negative Signif. %	24	17	37	0	50	13	0	10	27	70	13	36	75	75	30	100	68	7	27	35	35	33	57	0	6
Negative Insignif. %	21	11	12	25	25	9	33	11	9	0	21	33	5	0	5	0	16	28	27	21	23	33	33	25	15
**Negative Total %**	45	28	48	25	75	22	33	21	36	70	33	70	80	75	35	100	84	35	55	56	58	65	90	25	21
**Signif. % (included studies)**	62	72	68	25	75	80	56	59	91	80	67	48	95	96	90	100	81	44	55	61	51	51	62	50	70
**Signif. % (all studies)**	13	10	61	1	4	75	4	30	7	6	12	12	14	20	13	2	22	14	4	26	16	16	10	3	27

Positive and negative percentages are calculated as (number of votes)/(number of studies including the variable).

Abbreviations: HH Size = Household Size; # Rms= Number of rooms in house; HH Educ.= Household Education; Fem Educ.= Female Education; Male Educ.= Male Education; Female HH= Female Head of Household; Self Empl.=Self Employed; Agri. Lab.=Agricultural Laborer; Cas. Lab.=Casual Laborer; Soc. Marg.=Socially Marginalized Group; Credit Acc.= Access to Credit; Kero.Price= Price of Kerosene; Elec. Price=Price of Electricity; Wood Avail.=Wood Availability; LPG Avail.=LPG Availability; Elec. Avail.=Electricity Availability

References

Adkins E, Eapen S, Kaluwile F, Nair G, Modi V. 2010. Off-grid energy services for the poor: Introducing LED lighting in the Millennium Villages Project in Malawi. Energy Policy 38(2):1087-1097.

Amacher G, Hyde W, Joshee BR. 1992. The Adoption of Consumption Technologies under Uncertainty: A Case of Improved Stoves in Nepal. J Econ Dev 17(2).

Amacher GS, Hyde WF, Kanel KR. 1996. Household fuelwood demand and supply in Nepal's tarai and mid-hills: Choice between cash outlays and labor opportunity. World Dev 24(11):1725-1736.

Arthur MdFSR, Zahran S, Bucini G. 2010. On the adoption of electricity as a domestic source by Mozambican households. Energy Policy 38(11):7235-7249.

Chaudhuri S, Pfaff ASP. 2003. Fuel-choice and indoor air quality: a household-level perspective on economic growth and the environment. Department of Economics and School of International and Public Affairs, Columbia University.

Damte A, Koch SF. 2011. Clean Fuel Saving Technology Adoption in Urban Ethiopia. (Department of Economics Working Paper Series). Pretoria:University of Pretoria.

Edwards JHY, Langpap C. 2005. Startup Costs and the Decision to Switch from Firewood to Gas Fuel. Land Econ 81(4):570-586.

El Tayeb Muneer S, Mukhtar Mohamed EW. 2003. Adoption of biomass improved cookstoves in a patriarchal society: an example from Sudan. Sci. Total Environ 307(1-3):259-266.

Farsi M, Filippini M, Pachauri S. 2007. Fuel choices in urban Indian households. Environment and Development Economics 12(06):757-774.

Gebreegziabher Z, Mekonnen A, Kassie M, Köhlin G. 2010. Urban Energy Transition and Technology Adoption: The Case of Tigrai, Northern Ethiopia.

Gundimeda H, Köhlin G. 2008. Fuel demand elasticities for energy and environmental policies: Indian sample survey evidence. Energy Economics 30(2):517-546.

Gupta G, Köhlin G. 2006. Preferences for domestic fuel: Analysis with socio-economic factors and rankings in Kolkata, India. Ecological Economics 57(1):107-121.

Heltberg R. 2004. Fuel switching: evidence from eight developing countries. Energy Economics 26(5):869-887.

Heltberg R. 2005. Factors determining household fuel choice in Guatemala. Environment and Development Economics 10(03):337-361.

Hosier RH, Dowd J. 1987. Household fuel choice in Zimbabwe : An empirical test of the energy ladder hypothesis. Resources and Energy 9(4):347-361.

Jack DW. 2006. Household behavior and energy demand: Evidence from Peru [PhD Dissertation]. Cambridge: Harvard University.

Kavi Kumar KS, Viswanathan B. 2007. Changing structure of income indoor air pollution relationship in India. Energy Policy 35(11):5496-5504.

Kebede B, Bekele A, Kedir E. 2002. Can the urban poor afford modern energy? The case of Ethiopia. Energy Policy 30(11-12):1029-1045.

Kemmler A. 2007. Factors influencing household access to electricity in India. Energy for Sustainable Development 11(4):13-20.

Khandker SR, Barnes DF, Samad HA. 2010. Energy Poverty in Rural and Urban India: Are the Energy Poor Also Income Poor? (Policy Research Working Paper #5463).The World Bank.

Lamarre-Vincent J. 2011. Household Determinants and Respiratory Health Impacts of Fuel Switching in Indonesia [Master of Public Policy Thesis]. Durham: Duke University.

Louw K, Conradie B, Howells M, Dekenah M. 2008. Determinants of electricity demand for newly electrified low-income African households. Energy Policy 36(8):2812-2818.

McEachern M, Hanson S. 2008. Socio-geographic perception in the diffusion of innovation: Solar energy technology in Sri Lanka. Energy Policy 36(7):2578-2590.

Ouedraogo B. 2006. Household energy preferences for cooking in urban Ouagadougou, Burkina Faso. Energy Policy 34(18):3787-3795.

Peng W, Hisham Z, Pan J. 2010. Household level fuel switching in rural Hubei. Energy for Sustainable Development 14(3):238-244.

Pine K, Edwards R, Masera O, Schilmann A, Marrón-Mares A, Riojas-Rodríguez H. Adoption and use of improved biomass stoves in Rural Mexico. Energy for Sustainable Development In Press, Corrected Proof.

Rao MN, Reddy BS. 2007. Variations in energy use by Indian households: An analysis of micro level data. Energy 32(2):143-153.

Rebane KL, Barham BL. 2011. Knowledge and adoption of solar home systems in rural Nicaragua. Energy Policy 39(6):3064-3075.

Reddy BS. 1995. A multilogit model for fuel shifts in the domestic sector. Energy 20(9):929-936.

Walekhwa PN, Mugisha J, Drake L. 2009. Biogas energy from family-sized digesters in Uganda: Critical factors and policy implications. Energy Policy 37(7):2754-2762.

Wendland KJ, Pattanayak SK, Sills E. 2011. Democracy and Dictatorship: Comparing household innovation across the border of Benin and Togo. Raleigh, NC: North Carolina State University.

Yan HJ. 2010. The theoretic and empirical analysis on the compatibility of sustainable development strategies and poverty reduction policies at micro level. Aix-en-Provence: Université de la Méditerranée Aix-Marseille II.
